# HSULF-1 inhibits ERK and AKT signaling and decreases cell viability *in vitro* in human lung epithelial cells

**DOI:** 10.1186/1465-9921-13-69

**Published:** 2012-08-08

**Authors:** Huiying Zhang, Donna R Newman, Philip L Sannes

**Affiliations:** 1 Department of Molecular Biomedical Sciences, Center for Comparative Molecular Translational Research, College of Veterinary Medicine, North Carolina State University, Raleigh, NC 27606, USA; 2 Department of Environmental and Molecular Toxicology, College of Agriculture and Life Sciences, North Carolina State University, Raleigh, NC, 27606, USA

**Keywords:** Human endosulfatase 1, Heparan sulfate proteoglycans

## Abstract

**Background:**

Heparan sulfate proteoglycans (HSPGs) modulate the binding and activation of signaling pathways of specific growth factors, such as fibroblast growth factor-2 (FGF-2). Human endosulfatase 1 (HSULF-1) is an enzyme that selectively removes 6-O sulfate groups from HS side chains and alter their level and pattern of sulfation and thus biological activity. It is known that HSULF-1 is expressed at low levels in some cancer cell lines and its enhanced expression can inhibit cancer cell growth or induce apoptosis, but the mechanism(s) involved has not been identified.

**Methods:**

*HSULF-1* mRNA expression was assessed in five normal cells (primary human lung alveolar type 2 (hAT2) cells, adult lung fibroblasts (16Lu), fetal lung fibroblasts (HFL), human bronchial epithelial cells (HBE), and primary human lung fibroblasts (HLF)) and five lung cancer cell lines (A549, H292, H1975, H661, and H1703) using quantitative real time polymerase chain reaction (qRT-PCR). H292 and hAT2 cells over-expressing HSULF-1 were analyzed for cell viability, apoptosis, and ERK/Akt signaling, by MTT (3-(4,5-Dimethylthiazol-2-yl)-2,5-diphenyltetrazolium bromide) assay, TUNEL (Terminal deoxynucleotidyl transferase dUTP nick end labeling) assay, and Western Blot, respectively. Apoptosis pathway activation was confirmed by PCR array in hAT2, H292, and A549 cells.

**Results:**

HSULF-1 was expressed at a significantly lower level in epithelial cancer cell lines compared to normal cells. Infection with recombinant adenovirus for HSULF-1 over-expression resulted in decreased cell viability in H292 cells, but not in normal hAT2 cells. HSULF-1 over-expression induced apoptosis in H292 cells, but not in hAT2 cells. In addition, apoptosis pathways were activated in both H292 and A549 cells, but not in hAT2 cells. HSULF-1 over-expression reduced ERK and Akt signaling activation in H292 cells, which further demonstrated its inhibitory effects on signaling related to proliferation.

**Conclusions:**

These results indicate that HSULF-1 is expressed at lower levels in H292 lung cancer cells than in normal human alveolar cells and that its over-expression reduced cell viability in H292 cells by inducing apoptotic pathways, at least in part by inhibiting ERK/Akt signaling. We hypothesize that HSULF-1 plays important roles in cancer cells and functions to modify cell signaling, inhibit cancer proliferation, and promote cancer cell death.

## Background

Heparan sulfate (HS) proteoglycans are major components of extracellular matrix (ECM) and cell surfaces. They function as dynamic interfaces between cells and their external environment. They help cells affix to and maintain the extracellular scaffolding of the ECM as well as directly internalize lipid factors. Their shed ectodomain fragments can even neutralize injurious agents [1-4]. Importantly, they regulate essential cell signaling pathways which influence proliferation, differentiation, migration, and survival [3,5]. HS side chains are covalently linked to core proteins to form proteoglycans, such as syndecans or glypicans, which associate with or integrate into the lipid bi-layers of cells. HS side chains are also key structural features which facilitate ligand binding and receptor activation for an important group of signaling molecules which includes fibroblast growth factors (FGFs), wingless signaling glycoproteins (Wnts), Sonic hedgehog (Shh), hepatocytes growth factor (HGF), vascular endothelial growth factor (VEGF), and a newly defined tumor necrosis family member, a proliferation-inducing ligand (APRIL) [6-11]. For example, the loss of responsiveness to FGF by cells lacking endogenous heparan sulfate can be restored by the addition of exogenous soluble heparin [12]. Importantly, there is strong evidence that the nature of the sulfate bonds and the pattern distribution of sulfation are critical factors influencing signaling pathways [13,14]. FGF-2 signaling, for instance, requires the N- and 2-O-sulfate groups of heparan sulfate for binding to FGF-2 ligands and the presence of 6-O-sulfate groups for the activation of FGF receptor-1 (FGFR-1). Accordingly, 6-O-desulfated heparin, which binds to FGF-2 ligands but fails to bind to the FGFR, can decrease the FGF-2-induced proliferation of CHO677 cells [15].

Human endosulfatase-1 (HSULF-1) is the enzyme that specifically removes 6-O sulfate groups from HS side chains, thus modifying the pattern of sulfation and potentially changing its biological specificity. Although its quail analog QSULF-1 was found to be a secreted protein, HSULF-1 has been shown to be both secreted and localized on external cell surfaces as well as electrostatically attached to cell membranes [13,16,17]. Recently, the role HSULF-1 plays in cancer cell proliferation and embryonic development has been studied by several groups. The expression of HSULF-1 was found to be down-regulated in ovarian, breast, and hepatocellular cancers compared with normal epithelium [10,18], and its over-expression reduced tumor growth in several cancer types [19,20]. These collective observations support the notion that HSULF-1 plays an important role in the biology of some cancer cells. However, few studies have examined HSULF-1 activity in normal and cancer cells of the lung, the regulation of its expression, and its capacity to modulate lung cell proliferation and relevant signaling via hydrolyzation of 6-O sulfate groups. Accordingly, the aim of this study was to examine the expression of HSULF-1 and the effects of its over-expression in transformed human epithelial cells of pulmonary origin as compared with normal human alveolar type 2 cells.

## Materials and methods

### Cell preparation

H292, A549, HFL-1, and 16Lu cells were obtained from the American Type Culture Collection (*ATCC*) (Manassas, VA) and cultured in RPMI1640 (Roswell Park Memorial Institute 1640), F12K, and EMEM (Eagle’s minimal essential medium) media, respectively, with 10% FBS and antibiotics. Human AT2 cells and HLF cells were isolated from organ donor lungs obtained by the University of North Carolina Cystic Fibrosis/Pulmonary Research and Treatment Center Tissue Procurement and Cell Culture Core (Chapel Hill, NC). Isolated hAT2 cells were maintained in low-glucose DMEM (Dulbecco’s modified Eagle medium) medium supplemented with 10% FBS and Antibiotic-Antimycotic solution containing penicillin, streptomycin, and amphotericin B (Mediatech, Manassas, VA). Isolated HLF cells were maintained in high-glucose DMEM medium with 10% FBS and antibiotics. H1975, H661, and H1703 cells were purchased from Duke University’s Cell Culture Facility (Durham, NC) and maintained in RPMI medium with 10% FBS and antibiotics. HBE cells were gifts from Dr. Kenneth Adler and was maintained in Ham’s F-12/DMEM medium supplemented with 5 mg/ml insulin, 10 ng/ml epidermal growth factor, 0.1mM dexamethasone, 5 mg/ml transferring, 20 ng/ml cholera toxin, and antibiotics. The use of human cells was in compliance with the Helsinki Declaration, and approved by the North Carolina State University Institutional Review Board for the Protection of Human Subjects in Research.

### hAT2 and HLF cell isolation

Cells were isolated according to a scaled-up, modified version of the original Dobbs [21] procedure. In brief, an entire lobe from a donor cadaver lung was excised, cannulated, inflated to full capacity with Solution I, a PBS (phosphate buffered saline)-based solution lacking calcium and magnesium but containing EGTA, and lavaged and drained multiple times to remove macrophages, air and mucus. Divalent cations were restored by inflating with Solution II, a PBS-based solution containing magnesium and calcium. The lobe was inflated to capacity with elastase (Worthington Biochemicals, Lakewood, NJ) dissolved in Solution II (13 units/ml) and incubated at 37°C for up to forty minutes. Peripheral pieces were excised from the digested lobe and obvious bronchiolar tissue was removed and discarded. Tissue pieces were then minced into cubic millimeter size, using triple scissors, in Solution II containing DNase (Worthington). The minced tissue was transferred to a flask on ice and 5 ml of FBS was added to each 100 ml of suspension to neutralize the elastase. The suspension was shaken vigorously side-to-side in a 37°C water bath for 3 minutes to mechanically release the hAT2 and HLF cells from the tissue. The suspension was then filtered through a single layer of moistened cheesecloth several times until large pieces of undigested tissue were removed, then through two layers of cheesecloth twice and three layers once. The suspension was passed through 165 μm nylon mesh and finally through 42 μm nylon mesh. The filtrate was centrifuged at 1000 rpm (300 g) for 10 minutes at 4°C, and each cell pellet was re-suspended in 5 ml DMEM (without FBS) and pooled. A 100 μl aliquot of the cells was diluted 1:10 in a Trypan Blue solution and counted using a hemacytometer. Cells were re-suspended in sufficient DMEM so that around 20 million viable cells could be seeded on each of 40 Petri dishes coated with human IgG (Sigma, St. Louis, MO). Dishes of cells were incubated at 37°C for 1 hour to allow macrophages and white blood cells, as well as many fibroblasts, to adhere. Non-attached hAT2 cells were recovered by gently rocking each dish several times, transferred to 50 ml tubes, and centrifuged. Each hAT2 cell pellet was re-suspended in 5 ml DMEM and all cells were pooled in a single 50 ml tube. To further reduce fibroblast contamination, a mouse monoclonal anti-CD90/anti-fibroblast antibody, clone AS02 (EMD Biosciences, Inc., La Jolla, CA), was added to the cells for a 10 minute incubation at 4°C with gentle inversion. Excess antibody was removed by increasing the volume to 50 ml with DMEM/0.1% BSA and by pelleting the cells. The re-suspended cells (in 20 ml DMEM/0.1% BSA) were then incubated with pan-mouse-IgG Dynabeads for 30 minutes at 4°C with gentle inversion. The cell suspension was brought to 45 ml with DMEM/0.1% BSA and divided into three 15 ml tubes. Tubes were placed into a magnetic holder and the Dynabead-labeled fibroblasts were immobilized along the tube sides. The non-selected hAT2 cells were pooled in a 50 ml tube, counted, and plated in DMEM/10% FBS on rat-tail collagen-coated tissue culture dishes. After a medium change the next day, cells were cultured for 48 hours before further treatment. To obtain HLF cells, a portion of the mixed cell population which was not ASO2 depleted was placed on tissue culture dishes and cultured in complete medium until fibroblasts began to proliferate. These HLF cells were lightly trypsinized, transferred to flasks for amplification, and cryopreserved at passage 3. *Cell culture and treatment,* H292, A549, H1975, H661, H1703, HFL-1, 16Lu, HBE, HLF, and hAT2 cells were cultured for 48 hours and harvested for RNA analysis to measure the basal expression of *HSULF-1*. H292 and hAT2 cells were then cultured to 50% confluence and transduced with HSULF-1 adenovirus at 2, 5, 10, 20, 50, and 100 multiplicities of infection (MOI). After 24 or 48 hours, enzymatic conversion of formazan, an indirect measure of cell proliferation/viability, was measured by MTT assay. TUNEL assay was also utilized to confirm the cell death in transduced H292 cells over-expressing HSULF-1. In addition, H292, A549, and hAT2 cells, transduced at 10 MOI to over-express HSULF-1, were harvested for RNA and apoptosis pathway activation was analyzed by PCR array. Finally, selected signaling events influenced by over-expression of HSULF-1 were analyzed in H292 cells with or without 25 μg/ml heparin (Bovine lung, sodium salt, EMD Biosciences).

### Preparation of HSULF-1 adenovirus

Over-expression of HSULF-1 in epithelial cells was accomplished by adenoviral delivery of the human *SULF-1* gene driven by a CMV promoter. An Ultimate ORF (open reading frame) clone (IOH38422) in the pENTR221 vector (Invitrogen, Carlsbad, CA) was used to introduce the protein coding sequence of HSULF-1 into the pAd/CMV/V5-DEST vector (Invitrogen) by an LR Clonase II transfer and ligation reaction. The recombinant plasmid was transformed into TOP10 E. coli hosts and successful transformants were selected on Ampicillin plates. The HSULF-1 coding DNA was completely sequenced by primer walking to confirm 100% fidelity and a perfect clone was amplified and used to transfect 293A cells to produce adenovirus. Amplified adenoviruses were then titered by the Hexon antibody/DAB method and used to infect experimental hAT2 and H292 cells for transient over-expression of HSULF-1.

### MTT assay

A measure of cell proliferation/viability was obtained by a colorimetric assay which utilized the capacity of live cells to change 3-(4, 5-dimethylthiazol-2-yl)-2, 5-diphenyltetrazolium bromide (MTT) from yellow to a purple precipitate which could be dissolved in DMSO. Twenty-four, 48, or 72 hours after adenovirus infection of H292 and hAT2 cells, culture medium was discarded and the MTT solution (Sigma) was added to a final concentration of 1 mg/ml. After 3 hours of incubation at 37°C, the solution was removed and the formazan precipitate was dissolved in DMSO. Optical densities (OD) were measured at 570 nm using a microplate ELISA reader. Data was expressed as a percentage of untreated control cells and analyzed by ANOVA followed by Student’s *t*-test.

### TUNEL assay

Apoptosis was determined by TUNEL (Terminal deoxynucleotidyl transferase dUTP nick and end labeling) assay which detects the DNA fragmentation produced in apoptotic cells. Cells over-expressing HSULF-1 or lacZ (controls) were cultured for 72 hours, trypsinized, culture, washed, and re-suspended in 500 μl PBS. Each cell suspension was delivered into 5 ml of 1% paraformaldehyde. After incubation on ice for 15 minutes, cells were pelleted and supernatant discarded; cells were then washed twice in 5 ml of PBS and finally re-suspended in 500 μl PBS. This cell suspension was added to 5 ml of ice cold 70% ethanol, incubated on ice for 30 minutes, and pelleted again. Apoptosis was analyzed by a TUNEL kit (Invitrogen) which uses an anti-BrdU mouse antibody and Alexa Fluor 488 conjugate. Cells undergoing apoptosis exhibited a bright green nuclear fluorescence at excitation/emission wavelengths of 495/519 nm. Total cell nuclei were stained with DAPI (blue) and detected at excitation/emission wavelengths of 358/461 nm. Photomicrographs were taken of random fields with a Meiji MT6300H fluorescence microscope at 20X magnification with an Infinity 3 camera; DAPI and BrdU pictures were merged using Image J. A minimum of 3 fields was randomly selected and total cells were counted in each field to achieve a minimum number of 150 total. Apoptotic ratios as apoptotic cells/total cells are expressed as Mean ± standard error (SD) from different fields.

### Reverse transcription and quantitative real-time PCR

Cells were cultured as above for 48 hours and total RNA was isolated using the RNeasy Plus Mini Kit (Qiagen, Valencia, CA) according to manufacturer’s protocol. Four micrograms of RNA of each sample were reverse-transcribed utilizing the High-Capacity Archive Kit (Applied Biosystems, Foster City, CA). cDNAs were diluted to a final concentration of 10 ng/ml and 10 μl of each diluted sample (equivalent to 100 ng of RNA) were PCR-amplified in triplicate using Taqman primers and probes (Hs00392839_m1) (Applied Biosystems) on an iCycler (Bio-Rad, Hercules, CA). Data analysis was performed using the delta-delta CT method to compare the relative expression of *HSULF-1* normalized to GAPDH in different cell types; values and standard errors were graphed in Excel.

### PCR array analysis of apoptosis signaling pathways

hAT2, A549, and H292 cells were infected with lacZ or HSULF-1 adenovirus at 10 MOI for 48 hours. Cells were harvested and total RNA was isolated and purified by RNeasy Plus Mini Kit (Qiagen). Concentrations were measured spectrophotometrically at 260 nm and 1 μg of total mRNA was used as template for cDNA synthesis utilizing the High Capacity Archive Kit (Applied Biosystems). Produced cDNA was added to SybrGreen PCR master mix (SABiosciences, Frederick, MD) and aliquotted into each well of the ready-to-use PCR array PAHS-012 (Human apoptosis array, SABiosciences). Real-time PCR cycling was performed according to the protocol and data were analyzed using on-line programs from SABiosciences. The 84 apoptosis-related genes analyzed included tumor necrosis factor (TNF) ligand and receptor family, B-cell lymphoma 2 (BCL2) families, Caspases, Inhibitors of apoptosis (IAP), caspase recruitment domain family, death domain, death effector domain, and p53 family members.

### Preparation of cell lysates

Cells, cultured in 100 mm dishes, were rinsed with PBS. RIPA buffer (150 μl), containing PhosStop and Complete EDTA-free Protease inhibitors (Roche, Indianapolis, IN), was added to dishes. Cells were scraped and collected in microcentrifuge tubes and sonicated three times. Samples were then shaken on ice for at least 30 minutes and centrifuged at 14,000 rpm for 30 minutes at 4°C. Supernatants were transferred to fresh microcentrifuge tubes and total proteins in each sample were quantitated by Pierce 660 nm Protein Assay Kit (Pierce Biotechnology, Rockford, IL). The lysates were stored at -80°C.

### Western analysis

Equal amounts of proteins were subjected to electrophoretic separation in MOPS running buffer at 200V for 60 minutes on NuPage 4-12% Bis-Tris gels using the Novex X-Cell II system (Invitrogen). Proteins were transferred to nitrocellulose membranes and blocked with TBST/5% milk for 1 hour. Blocked membranes were incubated in Tris Buffered Saline containing 0.1% Tween20 and 5% BSA (TBS/T/BSA) with primary antibodies to HSULF-1 (Abcam, Cambridge, MA), p-ERK, ERK, p-Akt, Akt, and GAPDH (Cell Signaling Technology, Danvers, MA) overnight at 4°C with agitation. After washing in TBS/T, blots were incubated in TBS/T with 5% milk (TBS/T/milk) containing secondary antibodies conjugated to horseradish peroxidase (Cell Signaling Technology) for 2 hours with agitation. Bands on the membrane were detected by chemiluminescence using SuperSignal West Pico or Dura substrate (Pierce) and visualized by autoradiography. Integrated optical densities measured by ImageJ were exported to Microsoft Excel for analysis.

### Statistical analysis

Certain RT-PCR data were log transformed to obtain normally distributed variables. Values were expressed as mean ± SD and statistical significances were established by one or two tailed *t-test* and ANCOVA (analysis of covariance). A level of *p*<0.05 was considered significant.

## Results

### HSULF-1 basal expression is lower in lung cancer cells than in normal lung cells

**Figure 1:**
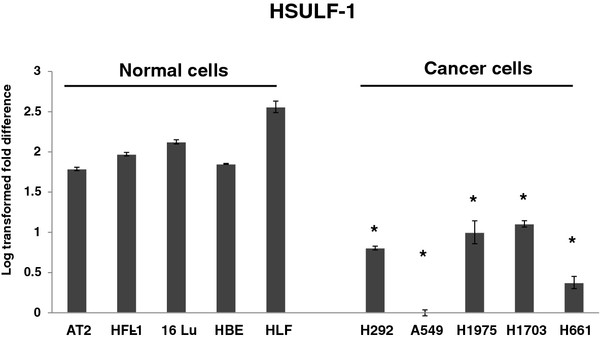
**Relative levels of baseline HSULF-1 expression.** Five normal cells (HFL-1, 16Lu, hAT2, HBE, and HLF) and five cancer cell lines (A549, H292, H1975, H1703, and H661) were cultured for 48 hours and the levels of HSULF-1 mRNA were analyzed by RT-PCR. Values were normalized to *HSULF-1* expression in A549 cells (lowest expression), log transformed to obtain normal distribution, and expressed as means ± SD from three separate analyses. All the cancer cells express HSulf-1 in a significantly lower level (*) than all normal cells at a p-value of 0.0004 as determined by one tailed Student’s *t*-test.

To evaluate the expression of *HSULF-1* in cells of pulmonary origin, five normal lung cells (fibroblasts (16Lu), fetal lung fibroblasts (HFL), primary lung fibroblasts (HLF), primary alveolar type 2 (hAT2) cells, and bronchial epithelial cells (HBE)) and five lung epithelial cancer cell lines (A549, H292, H1975, H661, and H1703) were cultured and mRNAs were analyzed. *HSULF-1* was expressed at a significantly higher level in normal lung cells (hAT2 (61-fold), HFL-1 (94-fold), 16Lu (133- fold), HBE (74-fold), and HLF (362-fold) compared to cancer cells (A549 as control, H292 (6-fold), H1975 (10-fold), H661 (2-fold), and H1703 (12-fold)) (Figure 1). This suggests that the expression of *HSULF-1* may be constitutively lower in lung cancer cells compared to normal cell lines or primary cells.

### Over-expression of HSULF-1 decreased cell density in H292 cancer cells but not in human primary hAT2 cells

**Figure 2:**
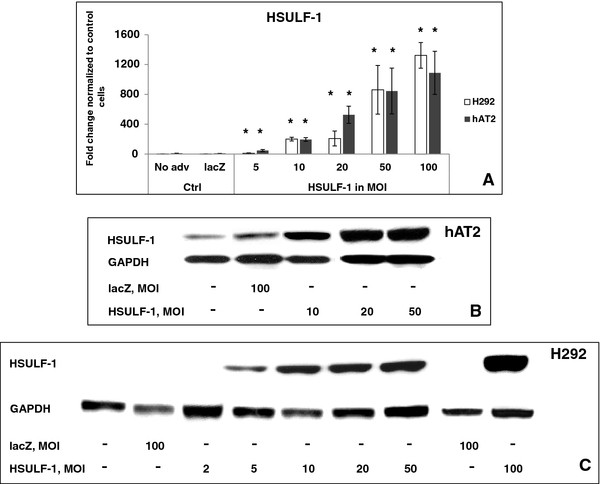
**Demonstration of *HSULF-1 *over-expression in hAT2 and H292 cells by quantitative real time PCR (qRT-PCR) and Western blot.** (**A**) Cells were infected with HSULF-1 adenovirus at MOIs from 5 to 100, with lacZ adenovirus at 100 MOI serving as a control. 48 hours post-infection, qRT-PCR was performed to assess the expression level of HSULF-1 mRNA. Values are shown as mean ± SD from 2 experiments. * Significant differences at p<0.05, compared with untreated and lacZ controls, are determined by Student’s *t*-test. Western blot was utilized to assess the expression level of HSULF-1 protein 72 hours post-infection in (**B**) hAT2 cells and (**C**) H292 cells.

**Figure 3:**
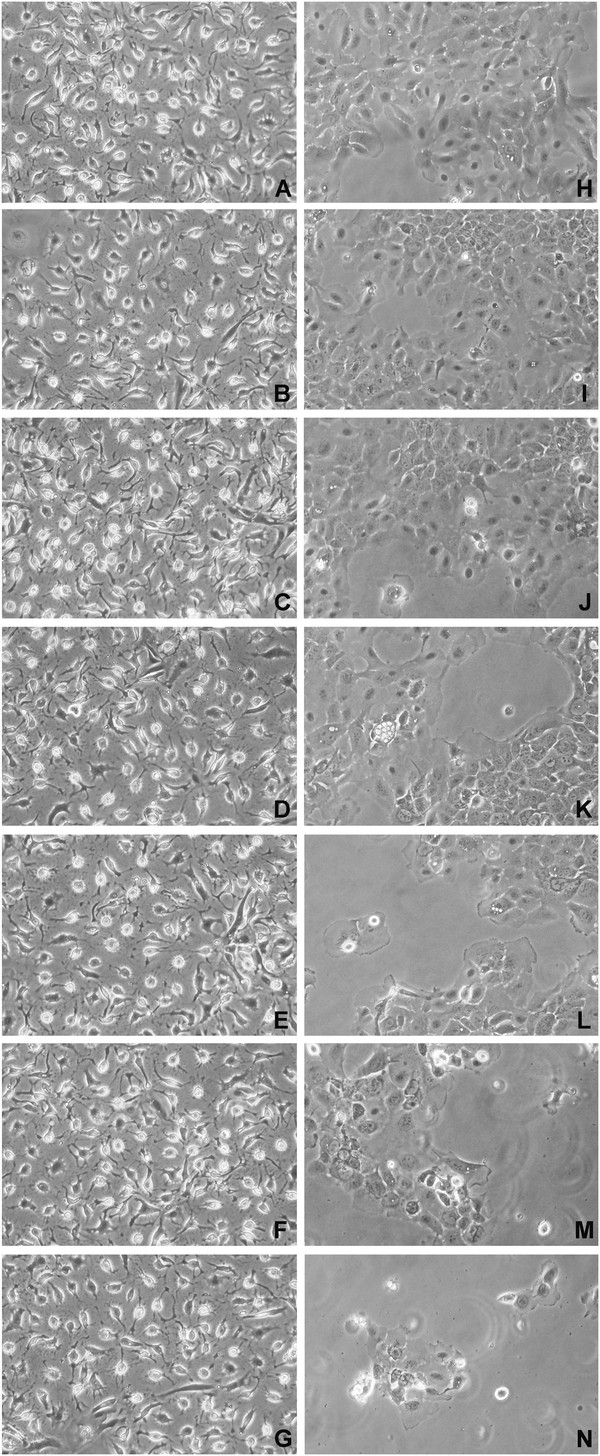
**Representative photomicrographs of hAT2 and H292 cells after over-expression of HSULF-1.** hAT2 (**B** – **G**) and H292 (**I** – **N**) cells were infected with HSULF-1 adenovirus with MOIs at 2, 5, 10, 20, 50, and 100 (top to bottom in sequence), with lacZ adenovirus at 100 MOI serving as control (**A** and **H**). Photomicrographs were taken after 72 hours under a phase contrast microscope at 200Х magnification.

H292 and hAT2 cells were infected with adenovirus at various MOIs for lacZ or HSULF-1 over-expression. Forty eight and 72 hours post-infection, quantitative real-time PCR and Western blot were performed to analyze the expression of HSULF-1 mRNA and protein, respectively. Results showed that the levels of HSULF-1 mRNA and protein were significantly increased (Figure 2, A – C). Seventy-two hours post-infection, phase-contrast microscopy showed that hAT2 cells infected with lacZ adenovirus at 100 MOI (FigureFigure 3A) were morphologically similar to untreated cells (data not shown), and were typically squamous in appearance with a centrally located nucleus. With increasing MOIs (2 – 100) of HSULF-1 adenovirus, hAT2 cells showed little or no significant change in morphology and density (Figure 3, B – G) compared with those infected with lacZ adenovirus alone. H292 cells infected with lacZ adenovirus were small, polygonal cells with a centrally positioned nucleus (Figure 3H) and morphologically similar to untreated cells (data not shown). In contrast to hAT2 cells, the morphology and cell density of H292 cells were altered by HSULF-1 adenovirus in a MOI-dependent manner. Low MOIs [2,5] did not significantly change the morphology or the cell density (Figure 3, I – J), while 10 MOI induced a visible decrease in cell density and an increase in the number of floating cells (Figure 3K). Higher HSULF-1 adenovirus MOIs (20, 50, and 100) not only induced morphologic changes in H292 cells but also decreased cell density (Figure 3, L – N).

**Figure 4:**
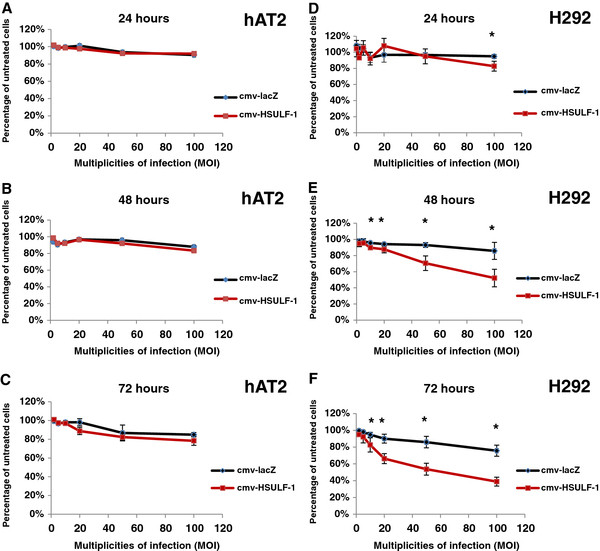
**MTT assay for hAT2 (A – C) and H292 (D – F) cells over-expressing HSULF-1.** Cells were infected with HSULF-1 adenovirus at 2, 5, 10, 20, 50, 100 MOIs with lacZ adenovirus as control at the same MOIs and cultured for 24, 48, or 72 hours. Values were normalized to untreated cells and shown as mean ± SD from 3 experiments. Significant differences compared with lacZ controls at p<0.05 are labeled by * as determined by ANCOVA and followed by Student’s *t*-test.

To quantitatively assess the viability of HSULF-1 over-expressing cells compared to lacZ over-expressing control cells, an MTT assay, which measures the activity of mitochondrial enzymes that reduce MTT to formazan and indirectly quantifies viable cells, was performed. In primary human alveolar hAT2 cells transduced with HSULF-1 adenovirus at 100 MOI, enzymatic activity was reduced to 90%, 83%, and 83% at 24, 48, and 72 hours, respectively, while hAT2 cells transduced with lacZ adenovirus at 100 MOI showed activity reduced to 92%, 87%, and 84%, compared to uninfected controls (Figure 4, A – C).

In H292 cells, 100 MOI of HSULF-1 adenovirus reduced MTT activity to 85%, 52%, and 39% at 24, 48, and 72 hours, respectively, while lacZ adenovirus reduced it to 95%, 88%, and 79% of uninfected controls, respectively. HSULF-1 also significantly reduced MTT activity at 10 to 50 MOIs in H292 cells at 48 and 72 hours compared to lacZ adenovirus infection (Figure 4, D – F). These results demonstrate that HSULF-1 over-expression lowers formazan conversion activity and thus indicates a reduction in viability in lung cancer cell lines, but not in normal lung cells.

### Over-expression of HSULF-1 induces apoptosis and related pathways in lung epithelial cancer cells

**Figure 5:**
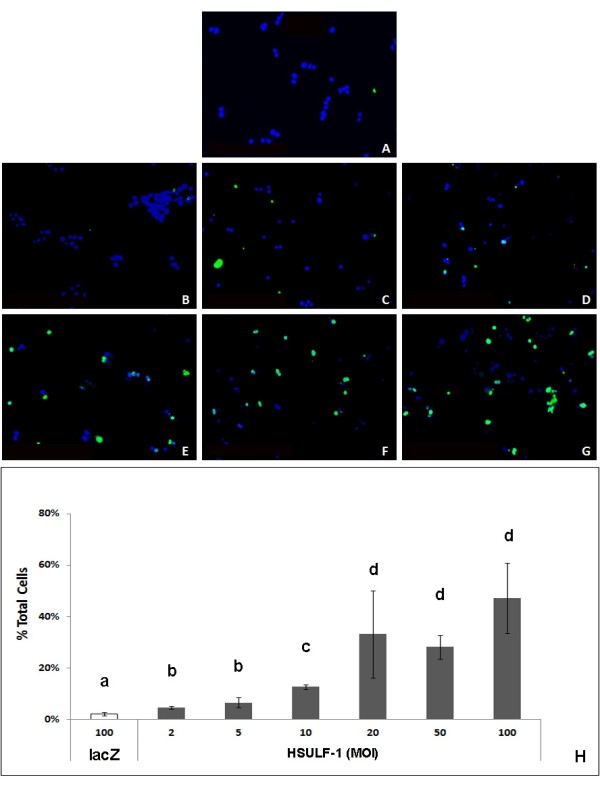
**TUNEL assay of H292 cells over-expressing HSULF-1.** Cells were infected with lacZ adenovirus alone (control, **A**) or HSULF-1 adenovirus at 2, 5, 10, 20, 50, and 100 MOIs (sequentially increasing, **B** – **G**). Seventy two hours post-infection, TUNEL assay was performed on harvested cells. Photomicrographs were taken of random fields and a minimum of 3 fields from photomicrographs were selected and total cells were counted in each field to achieve a minimum number of 150 total cells. (**H**) Apoptotic ratios as apoptotic cells/total cells are expressed as Mean ± SD from different fields. Significant differences between groups at p<0.05 as determined by Student’s *t*-test are indicated by the letters above each bar, with different letters indicating significant differences between groups.

To determine whether the reduced cell viability observed in the MTT assay was caused by apoptosis or by toxicity, lacZ or HSULF-1 over-expressing cells were subjected to TUNEL assay 72 hours after infection to assess DNA fragmentation as a quantitative measure of apoptosis. Results, confirmed in representative photographed fields, indicated that a high level of lacZ adenovirus (100 MOI) did not induce apoptotic cell death in H292 cells, with only rare co-localization of FITC-labeled foci (DNA fragments) with blue (DAPI) stained nuclei (Figure 5A). However, HSULF-1 over-expression did induce an increased number of FITC-labeled foci indicative of apoptosis in H292 cells, proportional to progressively increasing MOIs (Figure 5, B – G). Ratios of FITC-labeled foci to DAPI-labeled nuclei (total) indicated that HSULF-1 transduction, even at 5 MOI, induced significant apoptosis compared to lacZ control at 100 MOI, and higher MOIs of HSULF-1 adenovirus resulted in significantly greater apoptosis (Figure 5H).

**Figure 6:**
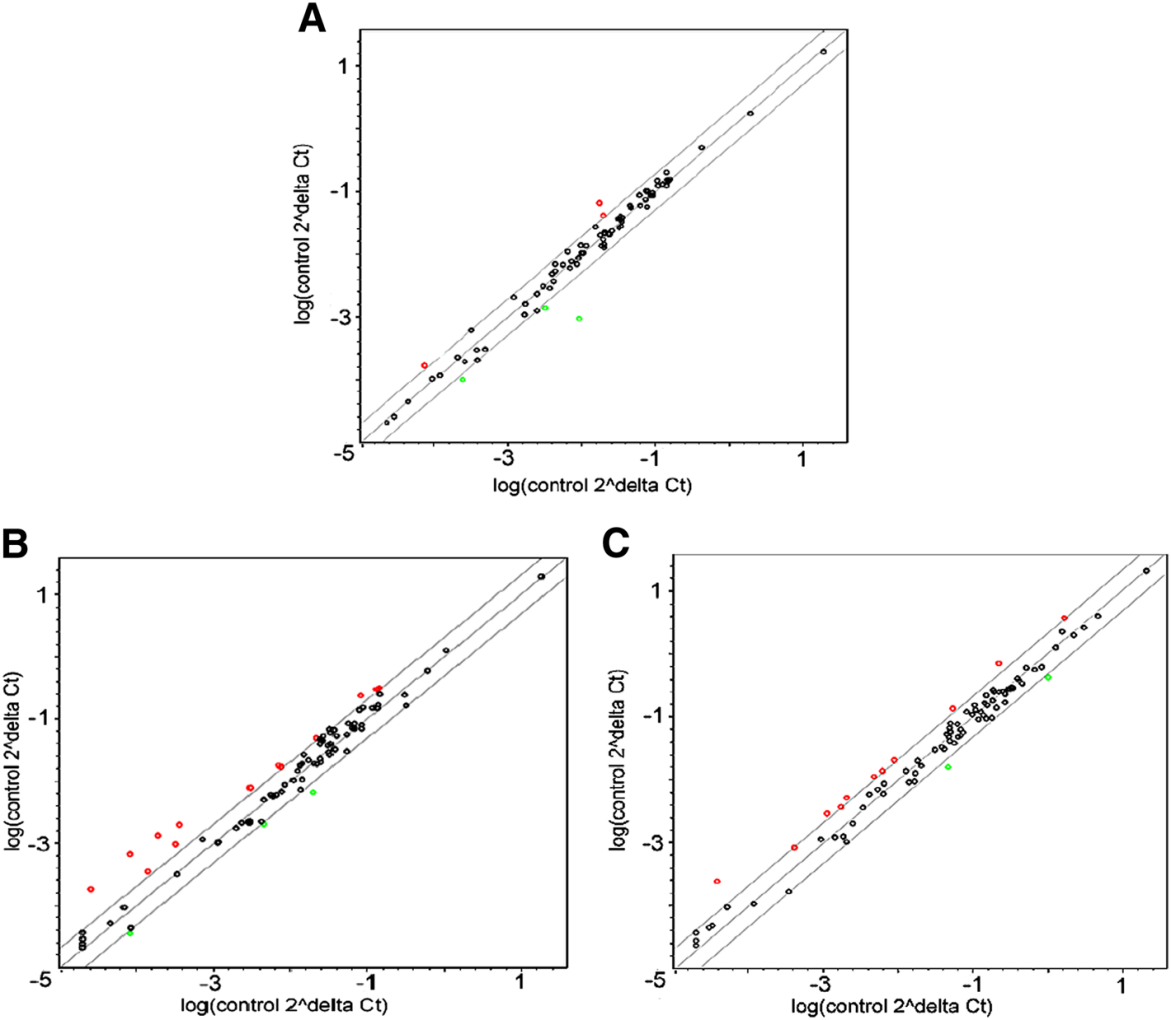
**Apoptosis pathway activation in hAT2 (A), H292 (B), and A549 (C) cells over-expressing HSULF-1.** At 48 hours post-infection with lacZ or HSULF-1 adenovirus at 10 MOI, mRNA was isolated and analyzed with an apoptosis PCR array. Genes significantly up- or down- regulated more than 2-fold compared to lacZ adenovirus controls are labeled red or green, respectively.

PCR arrays were then used to determine whether apoptotic signaling pathways were altered by HSULF-1 over-expression. Scatter plot analysis illustrated that data points representing activation of these apoptosis-related genes deviated less from those of lacZ adenovirus control in hAT2 cells (Figure 6A) than in H292 or A549 cells (Figures 6B and 6C) after over-expression of HSULF-1. Genes that were up- or down-regulated more than 2-fold (Table 1) revealed that in hAT2 cells, a total of six genes (*BCL2A1, BCL2L11, CASP7, DAPK1, IGF1R,* and *TNFRSF9*) were specifically activated by forced expression of HSULF-1. Of the four pro-apoptotic genes (*DAPK1, BCL2L11, CASP7,* and *TNFRSF9*), only *DAPK1* was down-regulated (-4.2-fold). The other three were up-regulated, but fold changes for two of these were close to 2. *TNFRSF9* was up-regulated (4.5-fold). The two anti-apoptotic genes (*BCL2A1* and *IGF1R*) were both down-regulated, but again close to the 2-fold change cut-off. This suggested that, on balance, there was insufficient activation of relevant pro-apoptotic pathways to support apoptosis compared to control cells.

**Table 1:** Genes that were up- or down-regulated in hAT2 cells by HSULF-1 over-expression

**AT2 cells**			
**Gene symbol**	**Description**	**Function**	**Fold change**
*BCL2L11*	*BCL2*-like 11 (apoptosis facilitator)	Pro-apoptosis	**2.1**
*CASP7*	Caspase 7	Pro-apoptosis	**2.1**
*DAPK1*	Death-associated protein kinase 1	Pro-apoptosis	**−4.2**
*TNFRSF9*	*TNF* receptor superfamily member 9	Pro-apoptosis	**4.5**
*BCL2A1*	*BCL2*-related protein A1	Anti-apoptosis	**−2**
*IGF1R*	Insulin-like growth factor 1 receptor	Anti-apoptosis	**−2.3**

hAT2 cells were infected with HSULF-1 adenovirus, total mRNA was isolated, and an Apoptosis PCR array was used to analyze gene expression. Genes that were significantly up- or down- regulated more than 2-fold compared to lacZ adenovirus control are shown.

In contrast, in H292 cells, eight pro-apoptotic genes (*BAX, CASP6, CASP8, CIDEA, DFFA, FAS, TNFRSF9,* and *TNFSF10*), four anti-apoptotic genes (*BAG1, BAG4, IGF1R,* and *BCL2A1*), and five unrelated genes (*GADD45A, CD70, TNFSF8, MCL1,* and *TP73*) were specifically altered by HSULF-1 expression. Of the eight pro-apoptotic genes, three (*CASP6, CASP8,* and *TNFSF10*) were down-regulated, but only *CASP6* by a relatively high amount (-4.5-fold). Five pro-apoptotic genes (*BAX, CIDEA, DFFA, FAS,* and *TNFRSF9*) were up-regulated, *CIDEA* (3.1-fold) and *TNFRSF9* (7.3-fold) more than the others, which were all close to the cut-off (Table 2). Of the four anti-apoptotic genes, three (*BAG1, BAG4,* and *IGF1R*) were slightly down-regulated and only *BCL2A1* was up-regulated at a relatively high fold-change (5.3-fold). Collectively, the up-regulation of the five pro-apoptotic genes combined with down-regulation of three anti-apoptotic genes would suggest that, on balance, apoptosis would be favored.

**Table 2:** Genes that were up- or down-regulated in H292 cells by HSULF-1

**H292 cells**			
**Gene symbol**	**Description**	**Function**	**Fold change**
*BAX*	*BCL2*-associated X protein	Pro-apoptosis	**2.1**
*CASP6*	Caspase 6	Pro-apoptosis	**−4.5**
*CASP8*	Caspase 8	Pro-apoptosis	**−2.1**
*CIDEA*	Cell death-inducing DFFA-like effector a	Pro-apoptosis	**3.1**
*DFFA*	DNA fragmentation factor	Pro-apoptosis	**2.2**
*FAS*	*TNF* receptor superfamily member 6	Pro-apoptosis	**2.3**
*TNFRSF9*	*TNF* receptor superfamily member 9	Pro-apoptosis	**7.3**
*TNFSF10*	*TNF* superfamily member 10	Pro-apoptosis	**−2.8**
*GADD45A*	Growth arrest and DNA-damage-inducible, alpha	Anti-proliferation	**2**
*CD70*	*TNF* superfamily member 7	Pro-proliferation	**6.6**
*TNFSF8*	*TNF* superfamily member 8	Pro-proliferation	**5.3**
*MCL1*	Myeloid cell leukemia sequence 1	Pro/anti-apoptosis	**2.1**
*TP73*	Tumor protein 73	Pro/anti-apoptosis	**2.5**
*BAG1*	*Bcl-2*-associated athanogene 1	Anti-apoptosis	**−2.3**
*BAG4*	*Bcl-2*-associated athanogene 4	Anti-apoptosis	**−2**
*BCL2A1*	*BCL2*-related protein A1	Anti-apoptosis	**5.3**
*IGF1R*	Insulin-like growth factor 1 receptor	Anti-apoptosis	**−2.2**

H292 cells were infected with HSULF-1 adenovirus, total mRNA was isolated, and an Apoptosis PCR array was used to analyze gene expression. Genes that were significantly up- or down- regulated more than 2-fold compared to lacZ adenovirus controls are shown.

Similarly, in A549 cells, ten pro-apoptotic genes (*APAF1, BAX, CASP3, CASP6, RIPK2, TNFRSF10B, TNFRSF25, TNFRSF9, TNFSF10,* and *LTBR*) and three anti-apoptotic genes (*BCL2A1, CD40LG,* and *XIAP*) were specifically altered by HSULF-1 over-expression. Of the ten pro-apoptotic genes, nine (*APAF1, BAX, CASP3, CASP6, RIPK2, TNFRSF10B, TNFRSF25, TNFRSF9,* and *TNFSF10*) were up-regulated and only *LTBR* was down-regulated. Of the three anti-apoptotic genes, two (*BCL2A1* and *CD40LG*) were up-regulated and *XIAP* was down-regulated. Collectively, the up-regulation of nine pro-apoptotic genes combined with down-regulation of one anti-apoptotic gene would suggest that apoptosis would be favored. This further supported the interpretation that over-expression of HSULF-1 reduced cell numbers through apoptosis in transformed H292 and A549 cells but not in hAT2 normal cells.

### Over-expression of HSULF-1 inhibits ERK and Akt signaling in lung cancer cell lines

**Figure 7:**
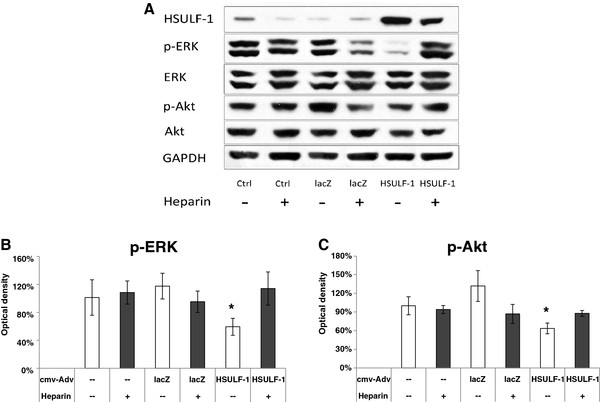
**ERK and Akt activation was inhibited by HSULF-1 but restored by heparin in H292 cells.** Cells were infected with lacZ or HSULF-1 adenovirus at 10 MOI, cultured for 48 hours, and exposed to 25 μg/ml heparin for 5 minutes. Top, Western blot analysis (**A**). Bottom, Densitometric analysis of p-ERK (**B**) and p-AKT (**C**). Integrated optical density values were analyzed using Image J, normalized to total ERK and Akt, and expressed as percentage of Control with means ± SD from 3 experiments. * Significant differences at p<0.05 compared to control cells, as determined by *t*-test.

It has been shown that HSULF-1 inhibits cell proliferation in several cancers and attenuates the activation of ERK and Akt signaling [8,9,22,23], which is maintained at a constitutively high level. The representative blot in Figure 7 compares activation of two key signal transduction pathway elements, p-ERK and p-Akt, in H292 cells untreated or transduced for lacZ or HSULF-1 over-expression. Western blot analysis indicates that lacZ over-expression slightly increased the levels of phosphorylated ERK and p-Akt, compared with untreated controls. Over-expression of HSULF-1 reduced the levels of phosphorylated ERK and p-Akt, compared to both the untreated and lacZ over-expression controls (Figure 7A). Densitometric analysis of p-ERK and p-Akt bands normalized against total ERK and Akt, respectively, indicated that over-expression of HSULF-1 significantly inhibited the phosphorylation of ERK (1%) and Akt (-38%) compared with untreated controls, and this inhibition was even more significant compared with lacZ controls (Figure 7, B – C).

To determine whether the signaling inhibition induced by HSULF-1 (i.e., loss of 6-O-sulfate groups) was reversible, a low, biologically relevant dose (25 μg/ml) of heparin, a model HSPG, was added to culture medium for 5 minutes. Heparin alone did not reduce the level of p-ERK or p-Akt in control cells (non-transduced) nor in lacZ over-expressing cells, but the inhibition of p-ERK and p-Akt with HSULF-1 over-expression was reversed to near control levels (Figure 7A). This was confirmed by densitometric analysis (Figure 7, B – C). These results indicate that the specific removal of 6-O-sulfate groups from extracellular HSPGs by HSULF-1 reduced the high level of constitutive ERK and Akt signaling in NSCLC cells, and this inhibition could be reversed by a brief pulse of sulfated heparin, which presumably counterbalanced the loss of sulfates and thus restored 6-O-sulfate-dependent signaling.

## Discussion

HSULF-1 is an important inhibitor of tumor/cancer cell growth [19] and is known to be down-regulated in various cancers, such as ovarian, head and neck squamous carcinoma, breast, gastric, kidney, and hepatocellular cancers [8-10,18,22-24]. One mechanism of HSULF-1 down-regulation is epigenetic silencing of its promoter region by hypermethylation [24]. It follows that down-regulation of HSULF-1 would enhance tumor growth, and it has been shown that over-expression of HSULF-1 in tumor cell lines inhibits specific, relevant signaling pathways dependent on growth factors including FGF-2, HB-EGF, HGF, and VEGF [9,10,13,18,23,25]. Lai, et al., demonstrated that reduced HSULF-1 expression in ovarian cancers resulted in an increased sulfated environment, which acted to enhance HB-EGF signaling and increase proliferation [18,20,23,26]. Another study found that HSULF-1 was down-regulated in several head and neck squamous carcinoma cell lines, and over-expression of HSULF-1 attenuated the activation of ERK/MAPK/Akt signaling stimulated by FGF-2 and HGF [8]. The negative regulation by over-expression of HSULF-1 on the FGF-2 signaling pathway is consistent with the fact that 6-O-sulfate groups are requisite for the binding of heparin to FGFR-1, which is necessary for forming a ternary complex with FGF2, and their removal would prevent this interaction [15]. These collective findings support the notion that down-regulating HSULF-1 provides cancer cells an environment sufficient in highly sulfated HSPGs, which act to promote selective growth factor signaling and attendant proliferation.

In this study, the goal was to determine the expression of *HSULF-1* in normal and transformed lung cells and its role in regulating cell signaling, survival, and apoptosis *in vitro*. Although investigated in several cell types recently, the role of HSULF-1 in pulmonary cells remains largely unknown. Results presented here demonstrate that *HSULF-1* expression is much lower in lung epithelial cancer cells (A549, H292, H1975, H661, and H1703) than normal cells (hAT2, HFL-1, 16Lu, HBE, and HLF) (Figure 1). This is consistent with previous studies showing that HSULF-1 down-regulation resulted in an environment that promotes proliferation [10,18,23,26].

To study the role of HSULF-1 in tumor growth, we chose to focus on its forced over-expression in H292 cells (a lung epithelial cancer cell line) compared with hAT2 cells. Consistent with previous findings, it was found that at 72 hours after adenovirally-mediated over-expression of HSULF-1, cell densities were visibly reduced in H292 cells in a concentration-dependent manner, but not in normal primary hAT2 cells (Figure 3). This was confirmed by MTT assay, which demonstrated that cell viability in H292 cells was significantly decreased by over-expression of HSULF-1 while hAT2 cells were unaffected (Figure 4). These results confirmed previous studies in which restored/enhanced expression of HSULF-1 reduced hepatocellular and breast cancer cell proliferation both *in vitro* and *in vivo*[18,22]. To further analyze the mechanisms of cell number reduction, TUNEL assay was performed on H292 cells and results showed that apoptosis was induced specifically by HSULF-1 over-expression (Figure 5). This is in agreement with previous studies that showed forced expression of HSULF-1 also increased the apoptosis induced by apicidin (a HDAC inhibitor) in Huh7 and Hep3B hepatocellular cell lines [9]. In addition, PCR array was utilized to analyze apoptosis activation at the gene level. Results showed that in H292 and A549 cells, more genes were activated, and more highly, by HSULF-1 over-expression than in hAT2 cells. Among them, the important cell death pathway effector BAX (induces mitochondrial leakage) was activated in H292 and A549 cells but not in hAT2 cells. Notably, important caspase family genes (*CASP3, CASP6, and CASP8*) and tumor necrosis family (TNF) genes (*TNFRSF10B, TNFRSF25, or TNFSF10*) were activated in H292 or A549 cells but not in hAT2 cells (Tables 1, 2, and 3, Figure 6).

**Table 3:** Genes that were up- or down-regulated in A549 cells by HSULF-1

**A549 cells**			
**Gene symbol**	**Description**	**Function**	**Fold change**
*APAF1*	Apoptotic peptidase activation factor 1	Pro-apoptosis	**2.1**
*BAX*	*BCL2*-associated X protein	Pro-apoptosis	**2.1**
*CASP3*	Caspase 3	Pro-apoptosis	**2.2**
*CASP6*	Caspase 6	Pro-apoptosis	**2.1**
*RIPK2*	Receptor-interacting serine/threonine-protein kinase2	Pro-apoptosis	**5.8**
*TNFRSF10B*	*TNF* receptor superfamily member 10B	Pro-apoptosis	**2.5**
*TNFRSF25*	*TNF* receptor superfamily member 25	Pro-apoptosis	**2.4**
*TNFRSF9*	*TNF* receptor superfamily member 9	Pro-apoptosis	**2.3**
*TNFSF10*	*TNF* superfamily member 10	Pro-apoptosis	**2.9**
*LTBR*	lymphotoxin beta receptor	Pro-apoptosis	**−2.7**
*BCL2A1*	*BCL2*-related protein A1	Anti-apoptosis	**2.4**
*CD40LG*	CD40 ligand	Anti-apoptosis	**2.0**
*XIAP*	X-linked inhibitor of apoptosis protein	Anti-apoptosis	**−2.2**

A549 cells were infected with HSULF-1 adenovirus, total mRNA was isolated, and an Apoptosis PCR array was used to analyze gene expression. Genes that were significantly up- or down- regulated more than 2-fold compared to lacZ adenovirus controls are shown.

These collective data suggest that normal cells may control their expression of HSULF-1 in order to modulate the surrounding sulfated environment to optimize responses to relevant growth factors. This is in agreement with previous work demonstrating that the optimal interactions of FGF-2 with their low affinity HSPG receptors, or heparin surrogates, lie within a relatively narrow range of concentration [27,28], and that these interactions are sulfate dependent [29]. This paradigm is shifted in cancer cells, wherein their requirements in a sulfated environment for maintenance and growth are high, making them very sensitive to reductions in sulfation, as seen with increased HSULF-1.

The impact of alterations in ligand binding events at the cell surface relating to FGF-2, VEGF, and HGF would be expected to be reflected in subsequent signaling pathways and account for the observed biological outcomes. It has been suggested that HSULF-1’s hydrolysis of sulfate groups from HSPGs down-regulates the receptor tyrosine kinase activity to attenuate cell growth and survival through signaling pathways regulated by FGF-2, VEGF, and HGF [19,30,31]. Based on these findings, H292 cells were forced to over-express HSULF-1 and levels of p-ERK and p-Akt, common down-stream targets of signaling pathways triggered by FGF-2, VEGF, and HGF, were analyzed. Results showed that p-ERK and p-Akt were inhibited by HSULF-1, and this inhibition was significantly reversed by restoring sulfated proteoglycans by addition of heparin (Figure 7). This is in agreement with previous studies showing that the loss of response of cells to FGF when lacking endogenous heparan sulfate can be restored by the addition of exogenous heparin [12]. Notably, both basal expression of p-ERK and p-Akt were high in the H292 cells compared with normal hAT2 cells (data not shown), which is typical of cancer cells [32,33] and may account for the heightened sensitivity to HSULF-1-induced reduction in signaling. A plausible mechanism for the apoptosis caused by HSULF-1 over-expression is that the significant reduction in p-ERK and p-Akt is sufficient to block proliferation and promote apoptosis and cell death in H292 cells, as the anti-tumor drug periplocin does in another NSCLC, A549, through its blockage of ERK and Akt pathways [34].

Interestingly, in contrast to inhibiting ERK and Akt signaling, HSULF-1 has been found to stimulate Wnt signaling and thus increase proliferation in pancreatic cancers [35,36], which reveals that the role of HSULF-1 is somewhat complicated. Recent studies found that *HSULF-1* is up-regulated in acute myeloid leukemia, pancreatic adenocarcinomas, T prolymphocytic leukemia, and in renal carcinoma, compared to corresponding normal tissues [37]. Furthermore, it has been shown that *HSULF-1* is expressed at higher levels in lung cancer patient samples compared with normal tissues, and high HSULF-1 expression could be associated with a poor prognosis in lung adenocarcinoma [37].

Several reasons to account for these apparent contradictions have been proposed. First, as shown in our experiments, the expression of HSULF-1 was compared in five lung cancer cell lines and five normal lung cells which were both randomly selected. After log transformation of the RT-PCR data, the results showed that the expression of HSULF-1 was significantly higher in normal cells than in cancer cells, with a p-value of 0.0004. This indicates that the wide variation in HSULF-1 expression and its effects may be explained in part by the differences between specific cancers and related cell lines and genetic variances in patient tissues. Second, in the study by Bret, et al., paired samples were obtained by surgical resection from lung squamous carcinoma and non-malignant neighboring tissues, which should contain different types of cells. Not only the cancer cells but also the cancer stromal cells may secrete HSULF-1, which may play different roles when produced by different cell types [37]. Also, the contributions of surrounding non-cancerous cells and a patient’s immune system to the local levels of HSULF-1 in an effort to combat a highly-aggressive cancer cannot be discounted. Thus, the up-regulation of HSULF-1 mentioned in lung squamous carcinoma may be explained partly by the increased expression in surrounding cells. Third, recently-described splice variants of Quail SULF-1 apparently have different functions, as the longer isoform A functions to enhance Wnt signaling while the shorter isoform B inhibits Wnt signaling but promotes angiogenesis [38]. The presence of SULF-1 alternate splicing forms has not yet been confirmed in humans, but it would be logical that a functional isoform could counterbalance or negate the function of the longer HSULF-1 or otherwise contribute to metaplasia in those human cancers over-expressing the isoform. Interestingly, and most recently, HSULF-1 gene polymorphisms were found to correlate with the age of onset, severity, and progression of ovarian cancer in a small study of human patients [39], adding yet another layer of complexity to be explored.

## Conclusions

Collectively, the present study reveals that HSULF-1 is expressed at lower levels in several lung cancer cell lines than in normal cells and its over-expression in H292 cells reduces cell viability and induces apoptosis by inhibiting ERK and Akt signaling. HSULF-1 plays important but complicated roles in cancer progression and inhibition depending on organ/tissue sites, cell types, environment, and those signaling pathways it affects. It should be viewed as an important target of cancer treatment.

## Abbreviations

Akt: Protein kinase B; AT1: Alveolar type 1 cells; AT2: Alveolar type 2 cells; ECM: Extracellular matrix; ERK: Extracellular signal-regulated kinase; FGF-1: Fibroblast growth factor 1; FGF-2: Fibroblast growth factor 2; FGFR: Fibroblast growth factor receptor; HFL-1: Human fetal lung fibroblast; HGF: Hepatocyte growth factor; HSULF-1: Human 6-O endosulfatase 1; HSPG: Heparan sulfate proteoglycan; lacZ: lac operon Z; MTT: 3-(4,5-dimethylthiazol-2-yl)-2,5-diphenyltetrazolium bromide; TGF-β: Transforming growth factor beta; TUNEL: Terminal deoxynucleotidyl transferase dUTP nick and end labeling; VEGF: Vascular epidermal growth factor.

## Competing interests

The authors declare that they have no competing interests.

## Authors’ contributions

HZ performed the experiments, collected and interpreted the data, and wrote the manuscript. DN prepared the adenoviruses and hAT2 cells, helped with protocols, and critically read the manuscript. PS planned the concept and design of the study and read and corrected the manuscript. All authors read and approved the final manuscript.
